# Blood Component Transfusion in Tertiary Care Neonatal Intensive Care Unit and Neonatal Intermediate Care Unit: An Audit

**DOI:** 10.7759/cureus.9952

**Published:** 2020-08-23

**Authors:** Rahulkumar J Amrutiya, Bhavdeep M Mungala, Viral T Patel, Jaishree D Ganjiwale, Somashekhar M Nimbalkar

**Affiliations:** 1 Department of Pediatrics, Pramukhswami Medical College, Karamsad, IND; 2 Department of Pediatrics, Aashirvad Superspeciality Children Hospital, Vadodara, IND; 3 Department of Pediatrics, Shree Krishna Children and Dental Hospital, Vadodara, IND; 4 Central Research Service, Bhaikaka University, Anand, IND; 5 Central Research Services, Bhaikaka University, Anand, IND

**Keywords:** blood component transfusions, neonates, international classification of diseases - 11 (icd-11), neonatal intensive care unit (nicu), fresh frozen plasma (ffp)

## Abstract

Background

Neonates admitted in a tertiary neonatal intensive care unit (NICU) require multiple blood transfusions because of extended NICU stay and repeated sampling. The rookie organ systems and miniature blood volumes in the neonate call for regular audits in neonatal blood transfusion practice. Sharing component usage data with the blood bank will prepare them to store components according to demand, thus limiting wastage of components as well as make banks ready to face a shortage in case of ramped up requirements.

Objective

Auditing neonatal blood transfusion indications and identifying the most commonly used component.

Methodology

This retrospective cohort study was conducted by the department of pediatrics over 22 months from February 20, 2017, to December 30, 2018. Any preterm and term neonates admitted to the NICU and Neonatal Intermediate Care Unit (NIMC) and receiving any transfusion, i.e., fresh frozen plasma (FFP), red cell concentrate (RCC), platelets, and exchange transfusion were included in our study. We collected data from the medical records of NICU and NIMC admitted patients receiving blood component transfusions from 2011 to 2016. Patients were categorized according to the classification of neonatal conditions by the International Classification of Diseases 11th Revision (ICD-11). There were no exclusion criteria. A descriptive statistical analysis was done, and a Chi-square test was applied.

Results

Out of 340 neonates, 249 (73.2%) were low birth weight, 139 (40.9%) were small for gestational age (SGA), and 277 (81.5%) neonates required transfusion during the first week of life. The majority of neonates require multiple transfusions. Fourteen(4.12%) neonates required up to 10 transfusions, two neonates required up to 22 transfusions, and 58 neonates required more than five blood transfusions. The majority required transfusion due to neonatal sepsis, Disseminated intravascular coagulopathy, low birth weight, respiratory distress syndrome, and unconjugated hyperbilirubinemia. Thirty-seven point eighty-two percent (37.82%) transfusions were fresh frozen plasma, 31.34% transfusions were red cell concentrate, 28.14% transfusions were platelet concentrate, and 2.70% were whole blood. Out of 340 neonates, 317 survived and were discharged.

Conclusion

The most commonly transfused component was fresh frozen plasma, the indication was neonatal sepsis, and the group was preterm. Whole blood is still being used and needs to be stopped.

## Introduction

Neonates admitted in a tertiary neonatal intensive care unit (NICU) require multiple blood transfusions as a consequence of extended NICU stay and repeated sampling. Neonate refers to an infant in the first 28 days after birth. A neonate's blood volume is tiny compared to the large transfusion bags they frequently receive [1]. For a 2.5 kg neonate, the total blood volume is approx 210 ml [2]. Multiple physiological changes occur when a fetus becomes a neonate. These changes refer to an alteration in their blood volume, hematological parameters, and other body systems. Stress to adapt in an extrauterine environment leads to a blunted capacity of premie to produce erythrocytes, thrombocytes, and immune cells, i.e., neutrophils. The blood volume in a full-term newborn is approximately 85 ml/kg while that in a preterm newborn is about 100 ml/kg and that in an adult is about 70 ml/kg. The miniature blood volume and rookie organ systems in the neonate call for novel proposals in neonatal blood transfusion practice [3]. The physiological puerility of different organ systems can put at peril those very low birth weight babies (VLBW) to metabolic imbalance following transfusion and their additives, and infectious and immunological hazards such as graft-versus-host disease (GVHD) [4]. However, with advanced transfusion medicine, the risk of getting infectious diseases and other adverse events related to blood transfusion is reduced significantly. Neonates are often transfused based on expert clinical opinion rather than specific documented guidelines [5]. Lack of statistically valid clinical trials put neonatal transfusion practice in controversy [6]. This study aims to delineate the neonatal blood transfusion indications and the most commonly used component. Sharing of component usage data with the blood bank will prepare them to store components according to demand, thus limiting the wastage of components as well as make banks ready to face a shortage in case of ramped up requirements.

## Materials and methods

This retrospective cohort study was conducted over 22 months from February 20, 2017, to December 30, 2018. The study was undertaken with approval by the Institute Ethics Committee. Any preterm and term neonates admitted to the NICU and NIMC at Shree Krishna Hospital, who received any transfusion, i.e., fresh frozen plasma (FFP), red cell concentrate (RCC), platelet concentrate (PC), and exchange transfusion, is included in our study. We have collected data from the medical records of NICU and NIMC admitted patients receiving blood component transfusions from 2011 to 2016. Table 1 shows the case study form used for data collection. Patients were categorized according to the classification of neonatal conditions by the International Classification of Diseases 11th Revision (ICD-11) [7]. There are no exclusion criteria. The analysis was done by descriptive statistics with the chi-square test. 

**Table 1:** Case study form

Serial No.	Blood Group of neonates
Name of Patient	Blood group of mothers
Age (in days)	Diagnosis
Gender	Blood component used (RCC/FFP/Platelet Concentrate/Exchange Transfusion)
Religion	Histogram and differential count: pre-transfusion and post-transfusion
Hospital No.	Volume transfused (approximately)
Date of Admission	The time between birth & transfusion received/day of transfusion
Type of Delivery	Complication due to transfusion
Birth Weight	Previous blood transfusion history
Preterm (<37 weeks)/Full term	Maternal diseases during pregnancy

## Results

We studied a total of 340 neonates, 256 (75.3%) were male and the rest were female. Out of 340 neonates, 249 (73.2%) were low birth weight. Two-hundred eighty-three (283; 83.2%) belonged to the Hindu community, which is predominant in the Anand locality. Two-hundred five (205; 60.3%) delivered via spontaneous vaginal delivery, whereas 135 (39.7%) delivered by cesarean section. The blood group of neonates was as follows: B+ 110 (32.4%) > O+ 109 (32.1%) > A+ 76 (22.4%) > AB+ 27 (7.9%), and the rest 18 neonates had a negative blood group. Twenty-one (6.2%) were twin pregnancies, and 319 (93.8%) were a singleton pregnancy. Three-hundred thirty-eight (338; 99.4%) cried immediately after birth. Three-hundred thirty-five (335; 98.5%) were delivered at the hospital. Two-hundred one (201; 59.1%) neonates were appropriate for gestational age (AGA), and 139 (40.9%) were small for gestational age (SGA). Out of 249 patients, 33 (9.7%) were extremely low birth weight, 77 (22.6%) were very low birth weight, and 139 (40.9%) were low birth weight. Two-hundred seventy-seven (277; 81.5%) neonates require transfusion during the first week of life. The majority of neonates require multiple transfusions. Fourteen (4.12 %) neonates required up to 10 transfusions, two neonates required up to 22 transfusions, and 58 neonates require more than five blood transfusions. Fifteen (15) out of 33 extremely low birth weight babies required ≥ five transfusions, 40 out of 139 low birth weight neonates required ≥ five transfusions, and 18 term neonates required ≥ five transfusions. A statistically significant correlation is found between neonatal birth weight and multiple transfusion needs (p = 0.01). Low birth weight neonates required more transfusions. The majority required transfusion due to neonatal sepsis, disseminated intravascular coagulopathy, low birth weight, respiratory distress syndrome, and unconjugated hyperbilirubinemia. No transfusion-related complications were observed in our study. Three-hundred seventy-nine (379) bags of fresh frozen plasma, 314 bags of red cell concentrate, 282 bags of platelet concentrate, and 27 bags of whole blood were transfused. Out of a total of 1002 transfusions, 37.82% transfusions were fresh frozen plasma, 31.34% transfusions were red cell concentrate, 28.14% transfusions were platelet concentrate, and 2.70% were whole blood. Out of 340 neonates, 317 survived and were discharged.

**Figure 1:**
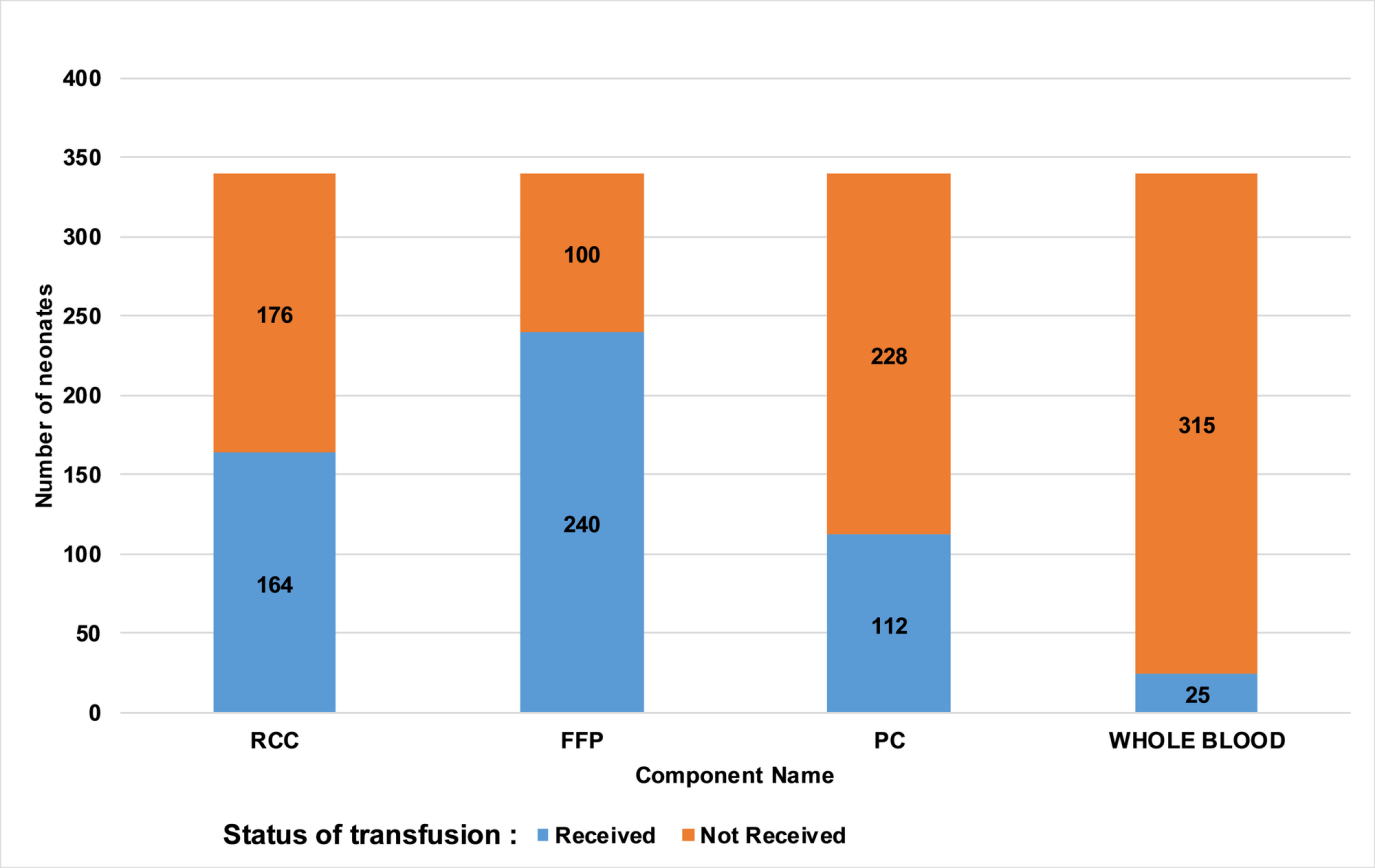
Graph showing the proportion of neonates who received component transfusion RCC = Red Cell Concentrate, FFP = Fresh Frozen Plasma, PC = Platelet Concentrate

Out of 340 neonates, 240 neonates received fresh frozen plasma (Figure 1). The pre-transfusion and post-transfusion comparison show significant improvement in neonates. Post-transfusion increase in hemoglobin, hematocrit, and platelet count was noted. Post-transfusion decrease in coagulation parameters was noted. Transfusion data for all of the components yielded a statistically significant result (Table 2).

**Table 2:** Changes in hematological parameters before and after blood transfusion *values in ×1,00,000 RCC = Red Cell Concentrate, FFP = Fresh Frozen Plasma, PC = Platelet Concentrate, WB = Whole Blood, PT = Prothrombin Time, aPTT = Activated Partial Thromboplastin Time, INR = International Normalized Ratio

Component name	No. of neonates	Mean	SD	P-value
Hemoglobin before giving RCC	154	12.35	3.65	<0.001
Hemoglobin after giving RCC	154	14.14	2.78	
Hematocrit before giving RCC	154	37.51	10.60	<0.001
Hematocrit after giving RCC	154	42.43	8.45	
Platelet count before giving PC	105	0.96*	0.95	0.004
Platelet count after giving PC	105	1.36*	1.26	
Hemoglobin before giving WB	22	12.95	3.42	>0.05 (0.770)
Hemoglobin after giving WB	22	13.55	2.22	
PT before giving FFP	189	33.74	15.77	<0.001
PT after giving FFP	189	18.7	8.8	
aPTT before giving FFP	231	81.67	30.04	0.002
aPTT after giving FFP	231	39.43	24.54	
INR before giving FFP	222	2.74	1.28	<0.001
INR after giving FFP	222	1.61	0.71	

Infections of the newborn is the category consuming the most red cell concentrate, fresh frozen plasma, and whole blood, whereas, hemorrhagic or hematologic disorders of the newborn received the most platelets (Table 3). Although the number of neonates receiving transfusions is higher, the analysis done in Table 3 only includes those neonates whose pre-transfusion and post-transfusion laboratory parameters were available.

**Table 3:** Classification of neonatal conditions according to ICD-11 and requirement of blood components ICD-11 = International Classification of Diseases 11th Revision, RCC = Red Cell Concentrate, FFP = Fresh Frozen Plasma, PC = Platelet Concentrate, WB = Whole Blood

Disease category according to ICD-11	RCC	%	FFP	%	PC	%	WB	%
Certain conditions originating in the perinatal period:								
Disorder of newborn related to the length of gestation or fetal growth	233	18.9	227	17	15	1.7	14	17.5
Respiratory disorder specific to the neonatal period	247	20	245	18.3	188	23.4	8	10
Cardiovascular disorder present in the neonatal period	38	3.1	59	4.4	71	8.8	2	2.5
Hemorrhagic or hematological disorders of newborn	162	13.1	194	14.5	205	25.5	20	25
Transitory endocrine or metabolic disorders specific to newborn	33	2.7	38	2.8	31	3.8	4	5
Disturbances of temperature regulation of newborn	6	0.5	5	0.4	4	0.5		
Infections of the newborn	266	21.6	259	19.3	75	9.3	19	23.8
Neurologic disorders specific to the perinatal or neonatal period	40	3.2	121	9	62	7.8	3	3.7
Digestive system disorders of the fetus or newborn	48	3.9	27	2	32	4	2	2.5
Certain disorders originating in the perinatal period; unspecified	15	1.2	16	1.2	9	1.2	1	1.3
Genitourinary system disorders specific to the perinatal or neonatal period	29	2.4	56	4.2	59	7.4	3	3.7
Diseases of the visual system	28	2.3	10	0.7	5	0.6		
Symptoms, signs or clinical findings, not elsewhere classified	26	2.1	21	1.6	22	2.8		
Developmental anomalies	61	5	61	4.6	26	3.2	4	5
Total	1232	100	1339	100	804	100	80	100

In our study, the top five conditions chewing blood products are sepsis (293), low birth weight (116), respiratory distress syndrome (93), neonatal hyperbilirubinemia (85), and disseminated intravascular coagulopathy (50) (Table 4).

**Table 4:** Few most frequent ICD-11 diagnoses ICD-11 = International Classification of Diseases 11th Revision

Broad diagnostic categories	Sub-categories	No. of patients receiving blood products
A) Certain conditions originating in the perinatal period		
a) Disorder of newborn related to the length of gestation or fetal growth	Low birth weight	116
	Very low birth weight	59
	Extremely very low birth weight	31
	Intrauterine growth retardation	4
b) Respiratory disorder specific to the neonatal period	Respiratory distress syndrome	93
	Birth asphyxia	78
	Apnea of newborn	44
	Neonatal aspiration of meconium	29
	Pulmonary hemorrhage	11
	Pneumothorax	11
	Pneumonia	10
	Bronchopulmonary dysplasia	5
	Transient tachypnoea of the newborn	2
c) Cardiovascular disorder present in the neonatal period	Persistent pulmonary hypertension of the newborn	40
d) Hemorrhagic or hematological disorders of newborn	Neonatal hyperbilirubinemia	85
	Disseminated intravascular coagulopathy	50
	Anemia of prematurity	28
	Intracerebral hemorrhage	9
	Blood incompatibility	6
	Acute bilirubin encephalopathy(kernicterus)	6
	Intraventricular hemorrhage	6
	Vitamin k deficiency	5
e) Transitory endocrine or metabolic disorders specific to newborn	Hypoglycemia	22
	Electrolyte imbalance	18
	Hyperglycemia	6
	Metabolic acidosis	4
f) Disturbances of temperature regulation of newborn	Hypothermia	8
g) Infections of the newborn	Sepsis	293
	Meningitis	32
h) Neurologic disorders specific to the perinatal or neonatal period	Hypoxic Ischemic Encephalopathy	76
	Neonatal Seizures	12
i) Digestive system disorders of the fetus or newborn	Gastro-oesophageal reflux disease	4
	Intestinal perforation	3
	Necrotizing enterocolitis	2
j) Certain Disorders originating in the perinatal period; unspecified	Underfeeding of newborn	12
	Failure to thrive	6
	Birth depression	5
k) Genitourinary system disorders specific to the perinatal or neonatal period	Acute Renal Failure	37
B) Diseases of the visual system		
a) Retinopathy of prematurity		10
C) Symptoms, signs or clinical findings, not elsewhere classified		
a) Multi-organ failure		11
D) Developmental anomalies		
a) Septal defects		26
b) Patent ductus arteriosus		16
c) Imperforate anus		2
d) Spina bifida		2
e) Renal agenesis		1
f) Down syndrome		1
g) Tracheoesophageal fistula		1
h) Meningomyelocele		1

Our study demonstrated that the rate of transfusion was higher in the neonate with a below-normal birth weight (Table 5). 

**Table 5:** Need for blood components in comparison to the birth weight of the neonate RCC = Red Cell Concentrate, FFP = Fresh Frozen Plasma, PC = Platelet Concentrate, WB = Whole Blood

Birth weight	Blood component			
	RCC	FFP	PC	WB
Normal Birth Weight	27 (16.5%)	72 (30%)	21 (18.8%)	8 (32%)
Low Birth Weight	58 (35.4%)	98 (40.8%)	46 (41.1%)	15 (60%)
Very Low Birth Weight	51 (31.1%)	47 (19.6%)	30 (26.8%)	1 (4%)
Extremely Low Birth Weight	28 (17.1%)	23 (9.6%)	15 (13.4%)	1 (4%)
Total	164 (100%)	240 (100%)	112 (100%)	25 (100%)

Table 6 shows that preterm neonates needed transfusions more often than full-term neonates. 

**Table 6:** Need for blood components in comparison to the gestational age of the neonate RCC = Red Cell Concentrate, FFP = Fresh Frozen Plasma, PC = Platelet Concentrate, WB = Whole Blood

Gestational age	Blood component			
	RCC	FFP	PC	WB
Preterm	108 (65.9%)	118 (49.2%)	69 (61.6%)	7 (28%)
Full term	56 (34.1%)	122 (50.8%)	43 (38.4%)	18 (72%)
Total	164 (100%)	240 (100%)	112 (100%)	25 (100%)

## Discussion

The results of the current audit highlight inconsistencies in practice vis-a-vis guidelines available. We show that many of the blood products given are at high levels of indications and readers are right in concluding that many were not needed. The audit is intended to bring a realization among practitioners in this field about the need to audit their own practices regularly. It is likely that due to various providers involved, many practices may not be in line with global recommendations. Using the quality improvement route may improve their practices in many areas.

Hematocrit is the primary determinant for transfusing RCC to a neonate. Opinion-based practice is favored widely in the absence of evidence-based protocols [6]. Many audits have identified several clinical scenarios, including the prevention of hemorrhage in critically ill neonates based on their coagulation profile. Neonatologists often transfuse plasma in the neonate with a deranged coagulation profile without any clue of active hemorrhage, despite a lack of data to prove the effectiveness of this practice [8]. Coagulation protein levels keep fluctuating depending on the gestational age of the neonate. This should be taken into account while interpreting prothrombin time (PT) and activated partial thromboplastin time (aPTT) [9]. Prolonged PT and aPTT are seen in premature infants due to the decreased synthetic function of hepatocytes. But prematurity itself is not an indication for transfusing fresh frozen plasma. Unless there is bleeding, FFP should not be used to restore a prolonged international normalized ratio (INR). Disseminated intravascular coagulation (DIC) without active bleeding does not justify the use of FFP. The use of FFP in liver disease is controversial, as you don't always get a full reversal of the coagulation defect [10]. Severe hyperbilirubinemia can be corrected rapidly with the use of whole blood exchange transfusion (WBET). WBET has the advantage of removing partially hemolyzed RBCs, antibody-coated RBCs, and circulating immunoglobulin [11]. One of the major causes of neonatal mortality is necrotizing enterocolitis (NEC) arising as a complication of red cell concentrate transfusions [12]. Blood transfusion practice depends solely on blood component availability, recipient’s epidemiology, and their response to therapy. A comparison between international transfusion practice differences and their impact on the recipient’s prognosis can help to construct a clear guideline for neonatal transfusion [13]. As compared to normal birth weight neonates, extremely low birth weight neonates required more frequent platelet transfusions when they were diagnosed with thrombocytopenia [14]. One cannot extrapolate adult transfusion data to fill gaps in neonatal transfusion cutoff [3]. Hemoglobin level in neonates differs from adults because they are age-dependent. Neonates have a hemoglobin level of 19 g/dl at birth, which dips down to 11.2 g/dl when they reach infancy. At one point in time, the child’s hemoglobin reaches 13 g/dl. At birth, 70% of the total blood is made up of fetal hemoglobin or HbF (and at six months of age, very less amount of HbF remains). In neonates, the oxyhemoglobin dissociation curve is shifted to the left secondary to shortened RBCs lifespan (90 days of neonate vs. 120 days of an adult). A higher blood transfusion/unit volume ratio in neonates threatens them to metabolic derangements with transfusion. These occur due to donors’ RBCs and preservatives used in blood bags. Hypothermia, hypocalcemia, hyperkalemia, acidosis, and hypomagnesemia are risks related to transfusion. Hyperkalemia arising due to blood transfusion poses a significant threat in the neonate and potassium levels should be actively monitored in neonate receiving >20 ml/kg transfusion volume (or lower if the neonate has kidney problems or hyperkalemia at the beginning of transfusion). Cardiac arrest is associated with large blood volume transfusion due to hyperkalemia, especially neonate receiving exchange transfusion. The risk for cardiac dysfunction is raised with hypocalcemia, as neonates have limited sarcoplasmic reticulum. Transfusion effects depend on the environment. Hypothermia can occur in neonates due to large body surface areas. Coagulopathy can be exacerbated by hypothermia [15].

Anemia poses a major health threat for extremely low birth weight babies. Among them, RCC transfusion was associated with increased mortality [16]. Preterm neonates, especially those born before 30 weeks are often anemic. The current study showed that 83.6% of RCC transfusions were done among below-normal birth weight babies. Gestational age and birth weight are inversely proportional to the number of RCC given [17].

Delayed cord clamping increases hemoglobin levels at birth, which may have a favorable effect on developmental outcomes [18]. The World Health Organization (WHO) recommends delaying cord clamping for a minimum of 30 seconds [19]. In comparison to early cord clamping, delayed cord clamping results in few RCC transfusions and a reduced chance of necrotizing enterocolitis and intraventricular hemorrhage compared with early cord clamping [20]. However, strict adherence to guidelines cannot be judged due to a lack of documentation about the time allowed before cord clamping. Improved outcomes occur with platelet transfusions for hemorrhage secondary to thrombocytopenia [21]. But, the majority of platelet transfusion in neonates are done prophylactically in a bid to prevent hemorrhage, even if the neonate is not actively bleeding [22].

Limitations

The study has several potential limitations. First, the data was obtained from one urban hospital, which may not accurately represent overall transfusion practices in India. A future prospective multicenter study, which incorporates hospitals in all states at different locations, will provide a better picture of the patterns of blood consumption throughout the country. Iatrogenic anemia occurring due to repeated blood sampling in the NICU is a major cause of anemia. Our hospital records lacked documentation regarding the number and amount of blood samples collected. So we cannot establish it as a cause of anemia that required RCC. Although this study presents data from a single tertiary care hospital and a small chunk of neonates who got a blood transfusion in India, the analysis of data provides a perception of the traits of blood transfusion recipients and generate the platform for planning more ecumenical blood utilization studies in India. This heralds more exploration in various settings targeted at yielding proof necessary to communicate policies and practices.

## Conclusions

Low birth weight and prematurity are the two risk factors identified that lead to multiple blood transfusions. Fresh frozen plasma was the most commonly transfused component, and the indication was neonatal sepsis. Whole blood is still used and must be avoided. Neonate’s varying body mass ratio, age-related physiology, immature heart, and weak immunity make transfusion requirements complex. This study presents a chance for measuring the use of and adherence to guidelines on prescribing blood in India. Findings from these types of audits will ensure a base for determining adherence to policies, guidelines, and practices in the proper clinical use of blood, leading to effective auditing and improvement in transfusion practices across neonatal intensive care units.

## References

[1] Keir AK, New H, Robitaille N, Crighton GL, Wood EM, Stanworth SJ (2019). Approaches to understanding and interpreting the risks of red blood cell transfusion in neonates. Transfus Med.

[2] Howie SRC (2011). Blood sample volumes in child health research: review of safe limits. Bull World Health Organ.

[3] Dogra K, Kaur G, Basu S, Chawla D (2018). Red cell transfusion practices in neonatal intensive care unit: an experience from tertiary care centre. Indian J Hematol Blood Transfus.

[4] Girelli G, Antoncecchi S, Casadei AM (2015). Recommendations for transfusion therapy in neonatology. Blood Transfus.

[5] (2019). Joint United Kingdom (UK) Blood Transfusion and Tissue Transplantation Services Professional Advisory Committee. https://www.transfusionguidelines.org/transfusion-handbook.

[6] Del Vecchio A, Franco C, Petrillo F, D'Amato G (2016). Neonatal transfusion practice: when do neonates need red blood cells or platelets?. Am J Perinatol.

[7] (2020). ICD - 11 (Foundation). https://icd.who.int/dev11/f/en.

[8] Keir AK, Stanworth SJ (2016). Neonatal plasma transfusion: an evidence-based review. Transfus Med Rev.

[9] Motta M, Del Vecchio A, Chirico G (2015). Fresh frozen plasma administration in the neonatal intensive care unit: evidence-based guidelines. Clin Perinatol.

[10] O'Shaughnessy DF, Atterbury C, Bolton Maggs P (2004). Guidelines for the use of fresh-frozen plasma, cryoprecipitate and cryosupernatant. Br J Haematol.

[11] Bujandric N, Grujic J (2016). Exchange transfusion for severe neonatal hyperbilirubinemia: 17 years’ experience from Vojvodina, Serbia. Indian J Hematol Blood Transfus.

[12] Marin T, Moore J, Kosmetatos N (2013). Red blood cell transfusion-related necrotizing enterocolitis in very low birth weight infants: a near-infrared spectroscopy investigation. Transfusion.

[13] Goncalez TT, Sabino EC, Capuani L (2012). Blood transfusion utilization and recipient survival at Hospital das Clinicas in São Paulo, Brazil. Transfusion.

[14] Curley A, Venkatesh V, Stanworth S (2014). Platelets for neonatal transfusion - study 2: a randomised controlled trial to compare two different platelet count thresholds for prophylactic platelet transfusion to preterm neonates. Neonatology.

[15] Palmieri TL (2017). Children are not little adults: blood transfusion in children with burn injury. Burns Trauma.

[16] Wang YC, Chan OW, Chiang MC (2016). Red blood cell transfusion and clinical outcomes in extremely low birth weight preterm infants. Pediatr Neonatol.

[17] Knee D, Knoop S, Davis AT, Rawson B, DiCarlo A, Olivero R (2019). Outcomes after implementing restrictive blood transfusion criteria in extremely premature infants. J Perinatol.

[18] Rabe H, Reynolds G, Diaz-Rossello J (2008). A systematic review and meta-analysis of a brief delay in clamping the umbilical cord of preterm infants. Neonatology.

[19] (2019). Delayed umbilical cord clamping for improved maternal and infant health and nutrition outcomes. Guideline. https://www.who.int/nutrition/publications/guidelines/cord_clamping/en/.

[20] Rabe H, Diaz-Rossello JL, Duley L, Dowswell T (2012). Effect of timing of umbilical cord clamping and other strategies to influence placental transfusion at preterm birth on maternal and infant outcomes. Cochrane Database Syst Rev.

[21] Galel SA, Fontaine MJ, Viele MK, Gonzalez L, Goodnough LT (2013). Transfusion medicine. Wintrobe's Clinical Hematology: Thirteenth Edition.

[22] Sola-Visner M (2012). Platelets in the neonatal period: developmental differences in platelet production, function, and hemostasis and the potential impact of therapies. Hematology Am Soc Hematol Educ Program.

